# Multiple patterns of EEG parameters and their role in the prediction of patients with prolonged disorders of consciousness

**DOI:** 10.3389/fnins.2025.1492225

**Published:** 2025-02-05

**Authors:** Hui Li, Linghui Dong, Wenlong Su, Ying Liu, Zhiqing Tang, Xingxing Liao, Junzi Long, Xiaonian Zhang, Xinting Sun, Hao Zhang

**Affiliations:** ^1^Cheeloo College of Medicine, Shandong University, Jinan, Shandong, China; ^2^China Rehabilitation Research Center, Beijing, China; ^3^University of Health and Rehabilitation Sciences, Qingdao, Shandong, China; ^4^Capital Medical University, Beijing, China

**Keywords:** prolonged disorders of consciousness, EEG, minimally conscious state, unresponsive wakefulness syndrome, microstate

## Abstract

**Introduction:**

Prognostication in patients with prolonged disorders of consciousness (pDoC) remains a challenging task. Electroencephalography (EEG) is a neurophysiological method that provides objective information for evaluating overall brain function. In this study, we aim to investigate the multiple features of pDoC using EEG and evaluate the prognostic values of these indicators.

**Methods:**

We analyzed the EEG features: (i) spectral power; (ii) microstates; and (iii) mismatch negativity (MMN) and P3a of healthy controls, patients in minimally conscious state (MCS), and unresponsive wakefulness syndrome (UWS). Patients were followed up for 6 months. A combination of machine learning and SHapley Additive exPlanations (SHAP) were used to develop predictive model and interpret the results.

**Results:**

The results indicated significant abnormalities in low-frequency spectral power, microstate parameters, and amplitudes of MMN and P3a in MCS and UWS. A predictive model constructed using support vector machine achieved an area under the curve (AUC) of 0.95, with the top 10 SHAP values being associated with transition probability (TP) from state C to F, time coverage of state E, TP from state D to F and D to F, mean duration of state A, TP from state F to C, amplitude of MMN, time coverage of state F, TP from state C to D, and mean duration of state E. Predictive models constructed for each component using support vector machine revealed that microstates had the highest AUC (0.95), followed by MMN and P3a (0.65), and finally spectral power (0.05).

**Discussion:**

This study provides preliminary evidence for the application of microstate-based multiple EEG features for prognosis prediction in pDoC.

**Clinical trial registration:**

chictr.org.cn, identifier ChiCTR2200064099.

## 1 Introduction

Prolonged disorders of consciousness (pDoC) encompass the vegetative state/unresponsive wakefulness syndrome (UWS) that characterized by an absence of conscious content, and the minimally conscious state (MCS) with discontinuous and fluctuating signs of conscious (Giacino et al., 2018; Schnakers, 2020). In clinical routine, both consensus-based expert diagnoses and widely used behavioral scale have been reported considerable misdiagnosis rates (Schnakers et al., 2009; Wannez et al., 2017). Identifying reliable features of conscious processing holds critical practical implications for prognostic discrimination.

Electroencephalography (EEG) provides objective information through multi-channel electrodes for studying and evaluating overall brain function and has been widely applied in pDoC (Bai et al., 2021). Since few patients with pDoC are able to follow clearly detectable commands (Cruse et al., 2011), resting-state spectral power and event-related potential (ERP) are two commonly used EEG detection technologies (Bai et al., 2021). These patterns reflect the spontaneous EEG signal oscillation (Piarulli et al., 2016) and processing abilities such as recognition and attention to sound of pDoC (Qin et al., 2008), respectively. However, these methods appear to be relatively insufficient for the comprehensive evaluation of continuous and dynamic complex brain functions (Demertzi et al., 2019).

Patients with impaired consciousness exhibit characteristics of global information processing deficits and increased local information processing in their brain networks (Rizkallah et al., 2019). Furthermore, as the level of consciousness decreases, the degree of integration within large-scale functional brain networks also diminishes (Panda et al., 2022). The temporal dynamics of these changes are particularly significant in pDoC (Panda et al., 2022; Panda et al., 2016). In recent years, numerous studies have reported that microstates may be associated with various psychological states (Baradits et al., 2020; Tamano et al., 2022; Bochet et al., 2021; Zanesco et al., 2020), providing insight into neural activity of the brain during resting-state. As a “quantitative indicator” of the distribution pattern of brain topographic maps, microstate analysis divides resting-state EEG signals into a limited number of distinct quasi-stable states (Khanna et al., 2015; Pascual-Marqui et al., 1995). Each microstate is distinctly associated with cortical regions of distinct brain networks described by neuroimaging methods. For example, state A is considered to be related to the auditory network, while state B is primarily activated in brain regions of the visual network (Custo et al., 2017). Although some studies have begun to explore changes in microstates of pDoC (Zhang et al., 2023; Li et al., 2024; Ling et al., 2024), research on their complete temporal characteristics remains limited, and more prognostic characteristics and underlying mechanisms need to be explained.

In addition, it is worth noting that previous studies have mostly relied on single EEG indicator or analytical method to assess the characteristics and prognosis of pDoC. Armanfard et al., reported that detecting of MMN component using machine learning can improve the accuracy of predicting the probability of recovery from coma (Armanfard et al., 2019). Sitt et al., jointly evaluated the diagnostic and prognostic value of spectral power and ERP for DoC (Sitt et al., 2014). However, to date, only a few studies have used quantitative indicators based on resting-state microstates to predict the prognosis of DoC (Stefan et al., 2018), and there is a lack of predictive models for different categories of indicators including microstates. Moreover, due to clinical heterogeneity and the lack of unified prognostic assessment criteria, the relative performance of these commonly used techniques in evaluating the prognosis of pDoC remains unclear.

The present study has two objectives: first, to explore the multiple EEG features of pDoC. Second, to develop predictive models using machine learning, evaluate the prognostic value of these indicators in a single dataset, and compare the importance of different patterns. To this end, we analyzed microstates, spectral power, and auditory evoked potentials from both patients with pDoC and healthy controls. Patients were further categorized according to their level of consciousness after 6 months. SHapley Additive exPlanations (SHAP) was introduced to identify the most important features in the model. This information will lead to a better understanding of neural function of human consciousness recovery.

## 2 Materials and methods

### 2.1 Patients and controls

This prospective study was undertaken at China Rehabilitation Research Center, Beijing, China. We consecutively recruited patients who met the definition of pDoC (Giacino et al., 2018) and were confirmed by the Coma Recovery Scale-Revised (CRS-R). Eligible patients were aged 18–75, had stable conditions, and with no history of mental illness, drug abuse, or open craniocerebral injury. Healthy participants were recruited from nursing staff and volunteers, who were native Chinese speakers without any mental or neurological diseases. All patients received at least 5 CRS-R assessments performed separately by 2 physicians within 1 week before enrollment to confirm a stable diagnosis of MCS or UWS (Wannez et al., 2017). The CRS-R includes 6 subscales involving auditory, visual, motor, verbal, communication, and arousal processes. Score of ≥3 for auditory, motor or verbal, or ≥ 2 for visual, or 1 for communication are consistent with a diagnosis of MCS. Lower scores represent a UWS (Giacino et al., 2004).

CRS-R was re-evaluated 6 months later followed the administration and scoring guidelines. Discharged patients were accompanied by family members to receive video consultation from professional doctors to check if they are in emergence from MCS (EMCS). EMCS are defined as recovering functional object use or communication from UWS or MCS (Giacino et al., 2002). Written informed consent was obtained from all healthy controls and the legal representatives of all patients before the study. The work was approved by the Ethics Committee of the China Rehabilitation Research Center. All procedures were conducted in accordance with the Code of Ethics of the World Medical Association (Declaration of Helsinki).

### 2.2 Procedures

EEG data were collected using a 32-channel electrode cap (NSM2, Neuracle, Changzhou, China). All participants took a short break of 1–2 min before the next block began. Firstly, 10 min of resting-state EEG were recorded. The ERP paradigm consisted of 2 blocks. Each block began with auditory presentation of the task instruction for that block: “The examination is about to begin. Please concentrate on the sounds”. In block1, a 1,200 Hz tone served as the deviant stimulus (20% of all stimuli), and an 800 Hz tone (80% of all stimuli) served as the standard stimuli. Stimuli were randomly delivered with a duration of 80 ms each and interval varying from 601 ms to 700 ms across 1,000 events. In block2, novel stimuli were presented as dog barking with a duration of 600 ms (10% of all stimuli), and 1,200 Hz and 800 Hz tones were 80 ms each, accounting for 20 and 70% of all stimuli, respectively. The time intervals randomly varied from 751 ms to 850 ms. The ground electrode was placed at Fpz on the scalp, and the reference electrode was placed at CPz. All electrode impedances were kept below 5kΩ, and the sampling rate was 1,000 Hz.

### 2.3 Data pre-processing

Recorded data were preprocessed using the EEGLAB toolbox under MATLAB (Li et al., 2024). After locating channels and removing unused electrodes, EEG data were bandpass filtered (high-pass: 0.1 Hz, low-pass: 45 Hz), followed by the use of a 48–52 Hz notch filter to suppress power line noise. Bad segments were then rejected, bad channels were identified and spherically interpolated. Artifacts including ocular and muscular activities were identified by independent component analysis. Specifically, resting-state EEG was divided into non-overlapping 2 s segments, and the average potential was calculated and re-referenced. ERP data were segmented into epochs of 500 ms for MMN and 600 ms from stimulus onset for P3a, including a 100 ms pre-stimulus period (Kruiper et al., 2019; Krugliakova et al., 2019), and re-referenced to the averaged mastoids.

### 2.4 Spectral power analysis

**Figure 1:**
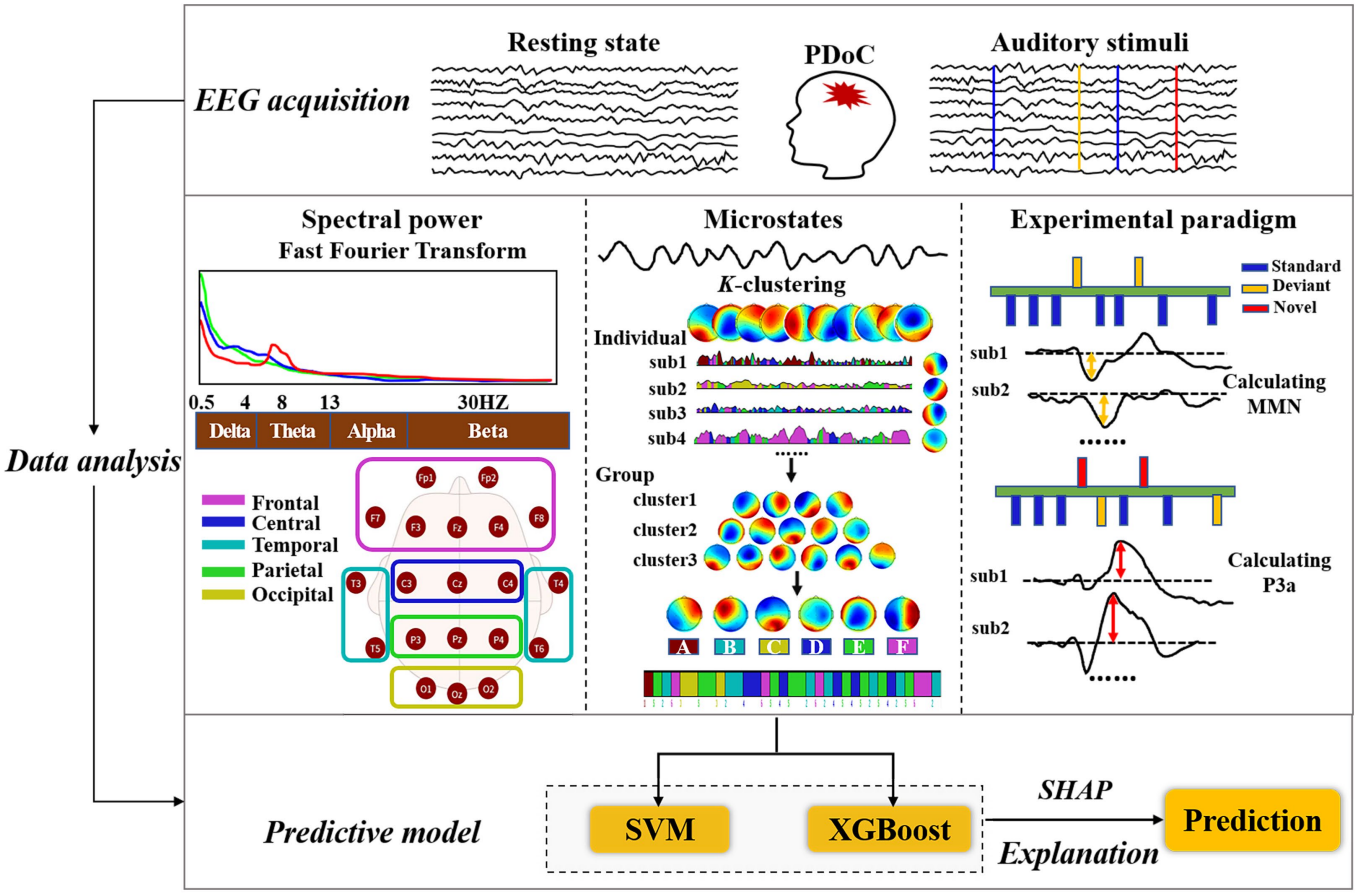
Diagram of EEG analysis in pDoC.

The spectral power of the selected EEG artifact-free epochs was analyzed using fast Fourier transform. Spectral power was averaged over the delta (0.5–4 Hz), theta (4–8 Hz), alpha (8–13 Hz), and beta (13–30 Hz) bands. The gamma was excluded due to potential contamination from high-frequency muscle movements (Rossi Sebastiano et al., 2015). Additionally, electrodes were grouped into five regions of interest (ROIs): Frontal (Fp1, Fp2, Fz, F3, F4, F7, F8), Central (Cz, C3, C4), Temporal (T3, T4, T5, T6), Parietal (Pz, P3, P4), and Occipital (Oz, O1, O2) (Babiloni et al., 2010) (Figure 1).

### 2.5 Microstate analysis

Microstate analysis was performed using the k-means clustering algorithm in Cartool EEG software (Damborska et al., 2019) following standard procedures (Baradits et al., 2020; Sun et al., 2021). To extract EEG microstates, the global field power was first computed by calculating the potential variance across all channels at each time point. All brain topographies at the peak sampling times of global field power were used for clustering. Custo’s early research identified seven microstates (Custo et al., 2017). Microstate A is characterized by strong activation in the left middle and superior temporal lobe. Microstate B simultaneously involves the left and right occipital cortical areas. Microstate C engages the precuneus and posterior cingulate cortex, while Microstate D involves the right inferior parietal lobe and right middle and superior frontal gyri. Microstates E, F, and G represent additional topographies distinct from the classical four states, displaying activation in the left medial frontal gyrus, dorsal anterior cingulate cortex, and right inferior parietal lobule, respectively. Previous studies have shown that a six-state classification can comprehensively capture the diversity of brain activity and elucidate the mechanisms of transition between different states, with each state corresponding to specific brain networks or functional modules, effectively reflecting the dynamic interactions within these networks (Damborska et al., 2019; Gold et al., 2022; Brechet et al., 2019).

In this study, we adopted a six-microstate classification and employed microstate segmentation to extract EEG features. Specifically, the identification of each topographic map was accomplished using Time-Aggregated Hierarchical Clustering (T-AAHC), an unsupervised learning algorithm typically used for clustering time series data (Haehner et al., 2023; Buetler et al., 2014). Initially, T-AAHC assumes that all raw maps from each subject constitute a single cluster, meaning the initial number of clusters equals the total number of raw microstate maps, denoted as n. Then, it identifies the cluster with the smallest variance explained ratio, considering it belongs to the same class as the cluster with the highest correlation coefficient. At this point, the number of clusters reduces to N−1. This process is repeated iteratively X times until N−X = 6, with the six clusters that explain the highest variance ratio taken as the individual clusters. Subsequently, all individual clusters from all subjects within each group are aggregated into a new set of raw maps, and the process is repeated to obtain common topographies.

By sorting individual EEG microstates and identifying common topographies for each group, we calculated the average group-level microstate classes for healthy controls, MCS and UWS patients. Group-average topographies were used to fit EEG clustering labels for individual subjects. The following microstate parameters were extracted: mean duration, coverage of time, occurrence per second and the transition probability (TP). Mean duration represents the average time (in milliseconds) that the microstate remains stable. Coverage of time measures the percentage of time covered by the EEG microstate category. Occurrence per second refers to the frequency of the microstate repeated per second. TP represent the probability of transitioning between brain microstates.

### 2.6 ERP analysis

Two ERP components evoked by independent blocks were identified: MMN in Oddball block 1 and P3a in block 2. Peak amplitude and corresponding latency were assessed using Matlab R2021b. After preprocessing the data with EEGLAB toolbox, MMN was represented as the ERP to the deviant stimuli subtracted from the ERP to the standard stimuli, calculated separately for each subject (Kotchoubey et al., 2005), and noted as the minimum amplitude within the window of 90 to 200 ms (Duncan et al., 2009). P3a was evoked by novel stimuli in block2 (Linnavalli et al., 2022), noted as the maximum amplitude within the window of 175 to 375 ms (Kruiper et al., 2019). Amplitude and latency of MMN and P3a components were calculated to obtain individual average values (Schnakers et al., 2008). For each component, only the data from the electrode Fz were analyzed for maximum amplitude and corresponding latency (Wu et al., 2020).

### 2.7 Machine learning analysis and feature importance

Python was used to conduct support vector machine (SVM) and extreme gradient boosting (XGBoost) for data analysis. According to patient’s prognosis (in EMCS or remain in pDoC), these two classification algorithms were developed to build predictive models of EEG features. SVM is a robust classification and regression model advantageous for small samples size, high dimensions, and accurate classification (Huang et al., 2023). XGBoost integrates and optimizes multiple decision tree models, offering high accuracy and interpretability (Wang et al., 2021). To maintain the correlation between different features, Min-Max normalization was applied to the raw data. The datasets for EMCS and pDoC groups were randomly divided into training sets (60% of the cohort) and test sets (40% of the cohort). Receiver operating characteristic (ROC) curves were plotted to evaluate the performance of each algorithm, and the optimal predictive model with higher area under the curve (AUC) was selected based on a comprehensive evaluation of EEG metrics. Predictive models for spectral power, microstates, and ERPs were further constructed to compare their contributions to prognosis.

To further interpret the importance of each feature in model prediction, SHAP values were introduced in this study. SHAP is a unified framework that effectively explains the underlying mechanisms and feature contributions of machine learning models (Wang et al., 2021). Therefore, we used SHAP to identify the top 10 factors of importance in the optimal model. In each feature importance plot, all patients’ attributions to the outcome are depicted as differently colored points, where red points indicating high SHAP values and blue points indicating low SHAP values. The higher the SHAP value of a feature, the more important it is for predicting EMCS.

### 2.8 Statistical analysis

Statistical analysis of the data was performed using SPSS software (version 27). For continuous variables, within-group comparisons were conducted using analysis of variance (ANOVA). Chi-square tests were used to examine differences in gender and etiology between groups. The Shapiro–Wilk test was used to assess data normality. Group was considered as a between-subjects factor, while bands (delta, theta, alpha, beta) and ROIs (frontal, central, temporal, parietal, occipital) as were considered as within-subjects factors for spectral power. Microstate classes (A-F) were analyzed as within-subjects factors for mean duration, coverage of time and occurrence per second of microstate. The latency and amplitude of MMN and P3a at Fz were analyzed. Age and gender were included as covariates. The Greenhouse–Geisser correction was applied for multiple comparisons. The significance level was set at 0.05 (with Bonferroni correction).

## 3 Results

### 3.1 Demographics

This study included 15 healthy people, 15 with MCS, and 15 with UWS (Table 1). There were no differences in gender and age between the three groups. No differences in the illness duration and etiology were found between patients in MCS and UWS. The CRS-R scores of MCS were higher than UWS. All participants completed a 10 min resting-state EEG examination and ERP testing. Six patients (20%) are in EMCS 6 months after EEG acquisition, 23 patients (76.7%) remained in pDoC, and 1 patient died of COVID-19. There were no differences in age, gender, etiology, course of illness, or CRS-R scores between the EMCS and pDoC groups (Table 2).

**Table 1:** Demographic and variables of MCS, UWS, and HC.

Variables	MCS	UWS	HC	Statistics	*p*-value
Categorical variables: (*N*)				Chi-square	
Gender (female/male)	5/10	7/8	7/8	0.729	0.695
Etiology	TBI (5)	TBI (10)	NA	3.652	0.143
	CVD (9)	CVD (5)			
	HIE (1)				
Continuous variables: mean (SEM)				ANOVA (F)	
Age (years)	52.4 (4.5)	47.2 (4.3)	37.9 (3.6)	3.107	0.055
				*t*-test (*t*)	
Illness duration (days)	276.8 (71.3)	219.7 (83.5)	NA	0.52	0.607
CRS-R	11 (0.6)	6.5 (0.5)	NA	6.054	<0.001

HC, healthy controls; MCS, minimally conscious state; UWS, unresponsive wakefulness syndrome; TBI, traumatic brain injury; CVD, cerebrovascular disease; HIE, hypoxic ischemic encephalopathy; CRS-R, coma recovery scale-revised; NA, no data.

**Table 2:** Variables of EMCS and PDoC groups in 6 months.

Variables	EMCS	PDoC	Statistics	*p*-value
Categorical variables: (*N*)			Chi-square (Fisher)	
Gender (*n*, female/male)	5/1	8/15	NA	0.064
Etiology (*n*)	TBI (3)	TBI (12)	0.623	1
	CVD (3)	CVD (10)		
		HIE (1)		
Continuous variables: mean (SEM)			*t*-test (*t*)	
Age (years)	46.8 (5.7)	50.2 (3.8)	−0.421	0.677
Illness duration (days)	111.8 (21.3)	287.1 (68.7)	−1.282	0.211
CRS-R	10.2 (1.1)	8.4 (0.7)	1.257	0.22

EMCS, emergence from MCS; PDoC, prolonged disorders of consciousness; TBI, traumatic brain injury; CVD, cerebrovascular disease; HIE, hypoxic ischemic encephalopathy; CRS-R, coma recovery scale-revised; NA, no data.

### 3.2 Spontaneous EEG oscillations

#### 3.2.1 Spectral power

**Figure 2:**
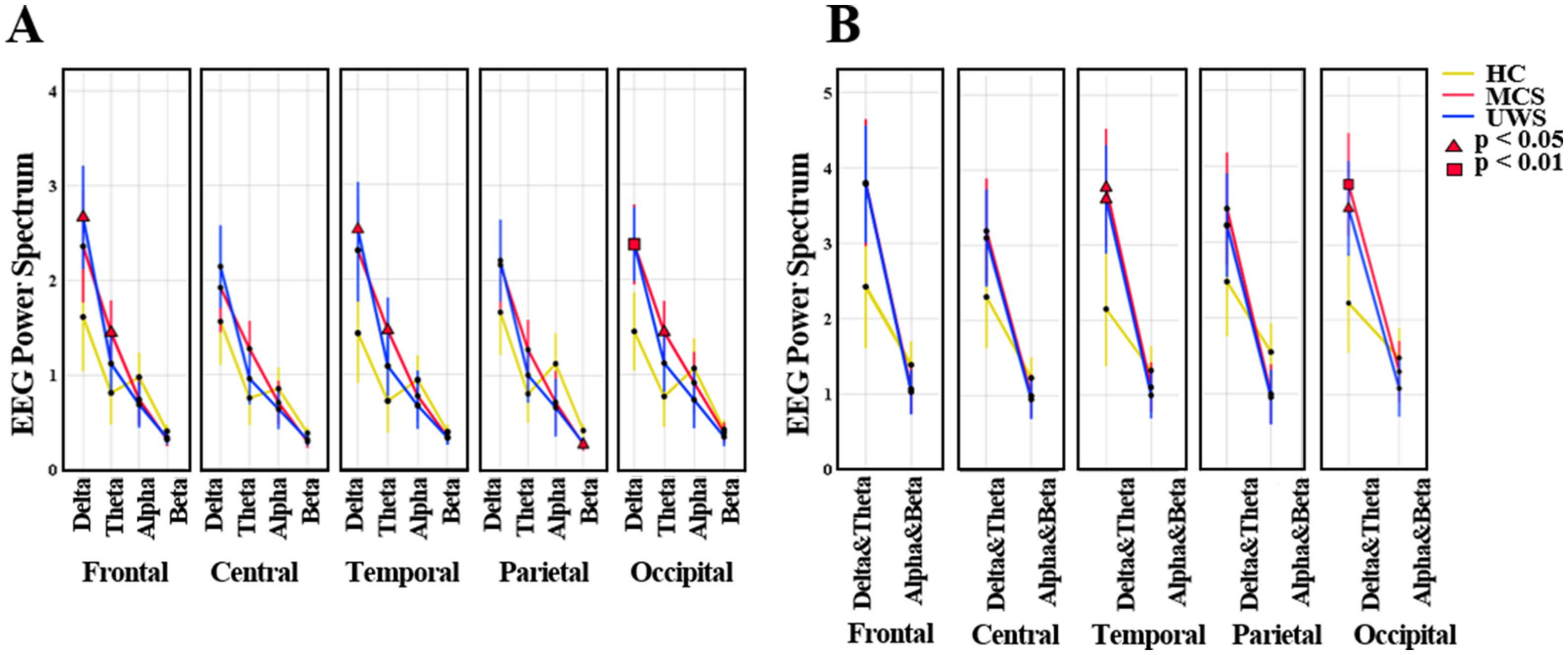
The average mean (95% CI) of EEG spectral power. **(A)** The values of frequency bands (delta, theta, alpha, beta) in HC, MCS and UWS groups. **(B)** The values of low (delta, theta) and high (alpha, beta) frequency bands in HC, MCS and UWS groups. HC, healthy controls; MCS, minimally conscious state; UWS, unresponsive wakefulness syndrome.

There was a statistically significant interaction between group (HC, MCS, UWS) and spectral power in band (delta, theta, alpha, beta) (*F* (3, 38) =7.549; *p* < 0.001). Figure 2 shows the average regional spectral power of the four frequency bands. Compared to healthy controls, UWS patients had higher delta power in the frontal (*p* < 0.05), temporal (*p* < 0.05), and occipital (*p* < 0.01) regions, while MCS patients had higher delta power in the occipital region (*p* < 0.05). MCS patients had higher theta power than healthy controls (frontal *p* < 0.05; temporal *p* < 0.05; occipital *p* < 0.05). Additionally, beta power in the parietal region was significantly lower in both MCS and UWS patients (*p* < 0.05).

#### 3.2.2 Microstates

**Figure 3:**
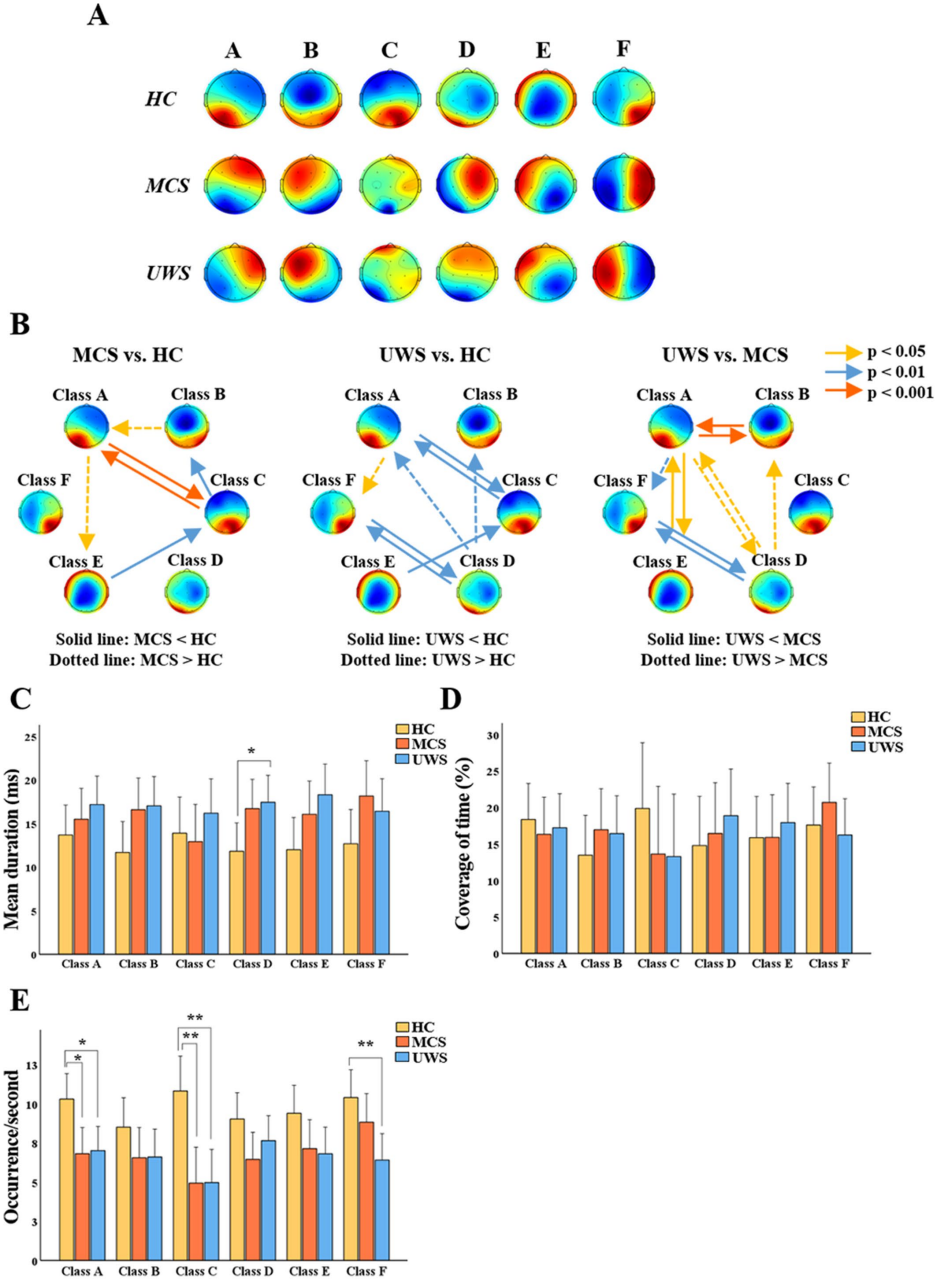
General properties of microstates. **(A)** Potential maps of the results of microstate segmentation in HC, MCS and UWS. **(B)** Comparison of the transition probabilities between HC, MCS and UWS. **(C–E)** Mean (95% CI) of the mean duration, coverage of time and occurrence number of six microstates among the three groups. HC, healthy controls; MCS, minimally conscious state; UWS, unresponsive wakefulness syndrome; * *p* < 0.05; ** *p* < 0.01 (Bonferroni-corrected).

The K-mean clustering algorithm provided six microstate maps (A, B, C, D, E, and F) for all study groups (Figure 3A). The results showed that compared to healthy controls, UWS had longer mean duration in class D (*p* < 0.05) and fewer occurrence number per second in class A (*p* < 0.05), C (*p* < 0.01), and F (*p* < 0.01). MCS had fewer occurrence number in class A (*p* < 0.05) and C (*p* < 0.01). No group differences were observed in terms of time coverage.

Compared to healthy controls, MCS showed significant reductions in TP between A and C (*p* < 0.001), from class C to B (*p* < 0.01) and E to C (*p* < 0.01), while TP increased from class B to A (*p* < 0.05) and A to E (*p* < 0.05). Furthermore, compared to healthy controls, UWS showed significant reductions in TP between class A and C (*p* < 0.01), D and F (*p* < 0.01) and from class E to C (*p* < 0.01), and increased TP from class D to A (*p* < 0.01), D to B (*p* < 0.01), and A to F (*p* < 0.05). In addition, compared to MCS, TP in UWS was lower between class A and B (*p* < 0.001), class A and E (*p* < 0.05), and class D and F (*p* < 0.01), while it was higher between class A and D (*p* < 0.05), A to F (*p* < 0.01) and D to B (*p* < 0.05) (Figure 3).

#### 3.2.3 MMN and P3a

**Figure 4:**
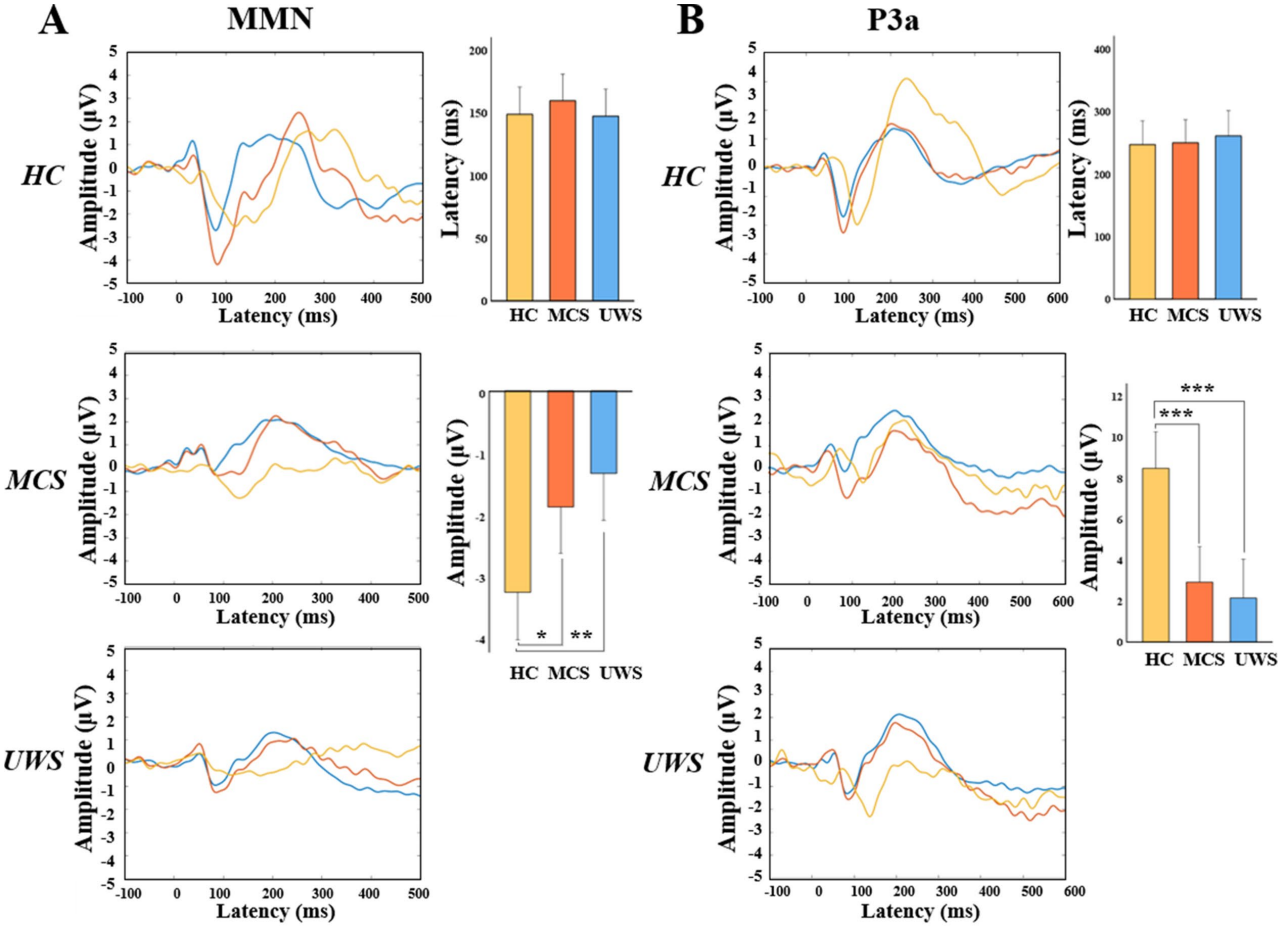
MMN and P3a amplitude on electrode Fz. Grand average waves and the statistical comparation of the averaged peak amplitudes and latencies (95% CI) over Fz electrode between HC, MCS and UWS groups of MMN **(A)** or P3a **(B)**. HC, healthy controls; MCS, minimally conscious state; UWS, unresponsive wakefulness syndrome. * *p* < 0.05; ** *p* < 0.01; *** *p* < 0.001 (Bonferroni-corrected).

Then we compared the amplitude and latency of MMN and P3a between healthy controls and patients with MCS or UWS. Results showed that there were no statistically significant differences in the average latency of MMN and P3a among HC, MCS, and UWS groups (148.93 vs.159.80 vs.147.50 ms, 246.80 vs.250.53 vs.261.08 ms). Amplitudes of MMN and P3a were significantly different among the three groups (*F* = 6.595, *p* = 0.003; *F* = 13.974, *p* < 0.001). Of which, the absolute amplitude of MMN was lower in MCS and UWS compared to healthy controls (*p* < 0.05, *p* < 0.01), and amplitude of P3a was significantly reduced in MCS and UWS (*p* < 0.001, *p* < 0.001) (Figure 4).

#### 3.2.4 Machine model development and feature importance

**Figure 5:**
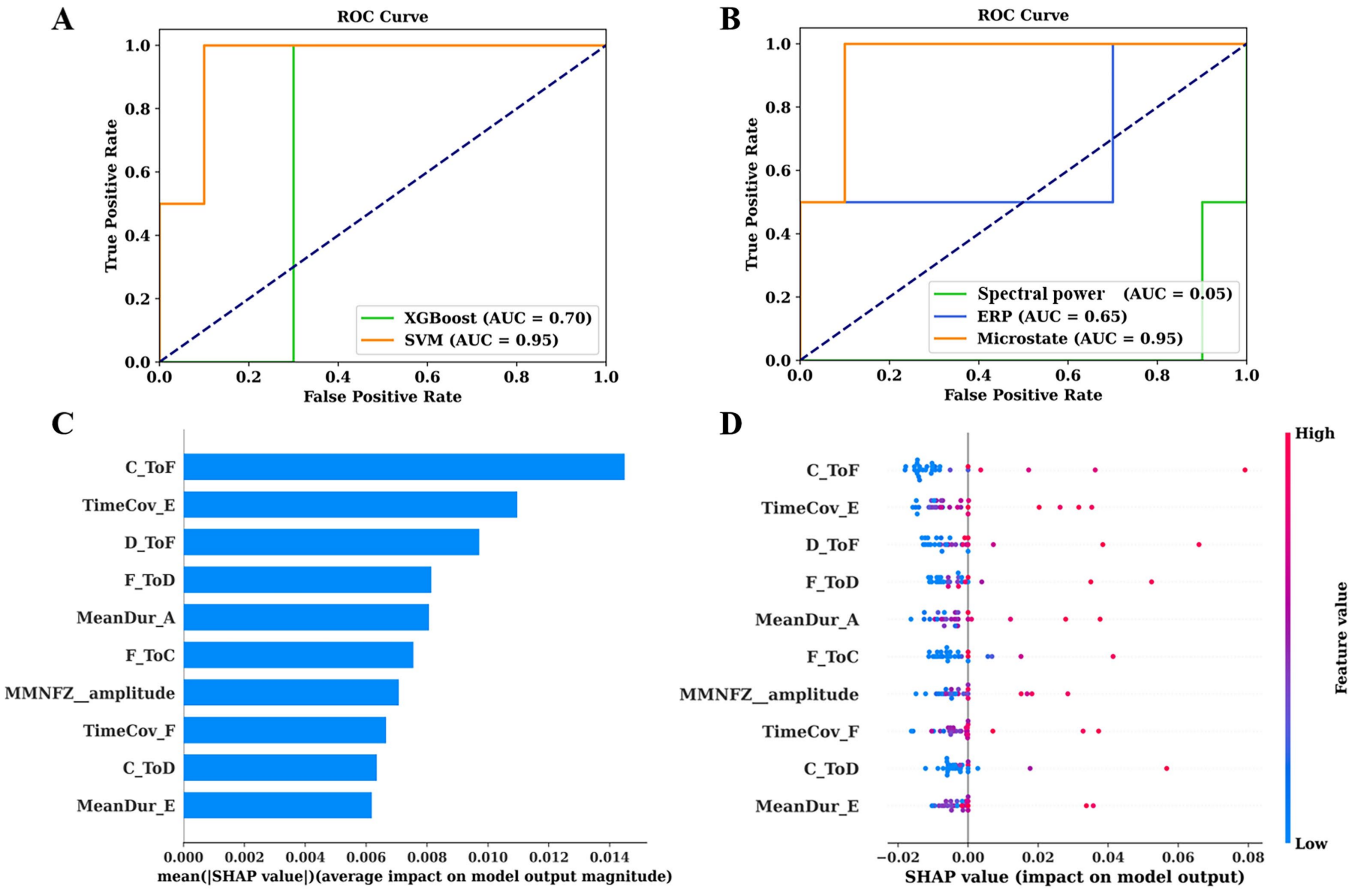
ROC curves of predictive models and their interpretation. **(A)** ROC curves of SVM and XGBoost for predicting prognosis of pDoC. **(B)** ROC curves of spectral power, microstates, and ERP for predicting prognosis using SVM. **(C)** The importance ranking of the top 10 features based on the mean absolute SHAP value. **(D)** The importance ranking of the top 10 features for stability and interpretation using SVM. The SHAP value (x-axis) is a unified metric that responds to the influence of the feature in the model. The feature ranking (y-axis) indicates the importance of the predictive model. ROC, receiver operating characteristic; AUC, area under the curve; SVM, support vector machine; XGBoost, extreme gradient boosting; SHAP, SHapley Additive exPlanations.

During the 6-month follow-up period, SVM achieved an AUC of 0.95 for prediction, which was significantly higher than that of XGboost (AUC = 0.7). Therefore, SVM was selected for subsequent prediction in this study. Furthermore, we used SVM to construct predictive models for spectral power, microstate, and ERP, respectively. Microstate showed the highest performance (AUC = 0.95), followed by ERP (AUC = 0.65), and finally spectral power (AUC = 0.05). To intuitively interpret the importance of each feature, we used Kernel SHAP to illustrate how these variables affect the overall predictive model. Figure 5C shows the top 10 risk factors evaluated by the average absolute SHAP value: TP from state C to F, time coverage of state E, TP from state D to F and D to F, mean duration of state A, TP from state F to C, amplitude of MMN, time coverage of state F, TP from state C to D, and mean duration of state E. Figure 5D shows its top 10 most important features associated with a higher predicted probability of EMCS in 6 months.

## 4 Discussion

This study explored the multiple EEG features of pDoC and developed a predictive model using SVM, achieving a high predictive performance (AUC = 0.95). By comparing the predictive capabilities of different modalities, we found that microstates contributed the most to the predictive model.

Prognostic prediction of pDoC is a complex and challenging task. Different EEG paradigms and analytical techniques convey different information, and the accuracy of the assessment may be influenced by confounding factors (Liuzzi et al., 2022). Therefore, it is particularly important to conduct evaluations using multiple methods. Previous studies have utilized EEG metrics for predicting the recovery of consciousness (Armanfard et al., 2019; Stefan et al., 2018; Liuzzi et al., 2022). We expanded on their work in the following aspects. Firstly, we comprehensively integrated commonly used EEG biomarkers (microstates, spectral power, and ERPs), attempting to provide additional prognostic markers through these features. When applying SVM to validate their predictive value, we obtained a higher AUC than previous studies, confirming the predictive value of multimodal EEG features (Figure 5A). Lastly, surprisingly, the results of SHAP values indicated that microstate parameters are highly significant for predicting EMCS (Figure 5C). We further compared the prognostic classification capabilities of different EEG patterns and confirmed that the model based on microstate data have the highest predictive ability (Figure 5B).

Spontaneous brain activity in the resting-state accounting for 80% of the entire energy consumption of the brain (Raichle and Mintun, 2006), especially concerning perception and consciousness (Wolff et al., 2019; Lee et al., 2022). In this study, we classified six microstates similar to the previous research (Zanesco et al., 2020; Damborska et al., 2019; Gold et al., 2022; Brechet et al., 2019) (Figure 3A). As mentioned, each resting-state topography is considered to activate certain cortical areas, representing synchronized activity of a distributed networks. Microstate A is closely related to the temporal lobe and involves auditory consciousness (Milz et al., 2016), while microstate B is associated with the occipital cortex (Britz et al., 2010). Microstate C is involved in information integration and the frontoparietal network (Li et al., 2024). Microstate D is an important electrophysiological representation of the attention network and is related to cognitive executive control functions (Michel and Koenig, 2018). Activation of microstate E is thought to be related to the default mode network that mediating internal consciousness (Li et al., 2023). Microstate F is spatially correlated with microstate C and plays a central role in the saliency network (Coste and Kleinschmidt, 2016; Sadaghiani and D'Esposito, 2015).

Transition from one microstate to another may represent sequences of network formations constituting large-scale brain networks (Baradits et al., 2020; Khanna et al., 2015). In this study, we found that MCS and UWS patients exhibited different TP compared to healthy controls (Figure 3B), indicating that the coupling and sequential activation of the corresponding brain networks and potential neuronal components are disturbed (Khanna et al., 2015). King et al., suggested that as the level of consciousness increases, the degree of information exchange between brain regions systematically increases, especially over longer distances in the cortex (King et al., 2013). The results here indicated that most transitions between microstates are more strongly correlated in MCS than in UWS and ranked high in the feature importance of predictive model (Figures 5C,D). Thus, enhanced activation and interaction between microstates are generally associated with better outcomes in pDoC (Wu et al., 2015). Moreover, our results suggest that transitions in brain networks or microstates related to consciousness are not merely one-way, unidirectional changes but involve simultaneous increases or decreases in both directions (Figure 3C). For example, MCS patients show stronger transitions between state F and state D compared to UWS patients (Figure 3B). These states activate regions that are densely interconnected via neural fibers, working together to influence attention allocation and executive control abilities (Kim et al., 2018).

The average duration of microstates reflects the stability of their underlying neural components (Khanna et al., 2015), while the occurrence frequency represents the tendency of activation of potential neural generators. Artoni et al., observed that the temporal dynamics and complexity of microstates increased with the depth of sedation (Artoni et al., 2022), leading to an obvious “U-shape” curve. These changes in microstate parameters indicate variations in the active regions of brain networks of pDoC (Figures 3C,E), which can also be validated by calculating the TP of microstates. With the recovery of consciousness, the orderliness of organizational function may increase and the randomness may decrease, providing new inspiration for future treatment.

In this study, we used two independent paradigms to calculate the MMN and P3a. Compared to healthy controls, both MCS and UWS patients had reduced amplitudes of MMN and P3a (Figures 4A,B), indicating a decline in discriminative ability and additional attentional capacity to sounds in pDoC patients (Kotchoubey et al., 2005; Linnavalli et al., 2022; Wang et al., 2018). Moreover, MMN also ranked in the top 10 important features in the prediction model (Figures 5C,D), confirming that auditory paradigm evoked potentials have certain predictive values for pDoC (Buetler et al., 2014). Additionally, consistent with previous studies (Piarulli et al., 2016; Rossi Sebastiano et al., 2015), we observed increased low-frequency band power in patients with pDoC (Figures 2A,B). Although previous studies have reported some predictive value of alpha power for consciousness recovery (Stefan et al., 2018), in this study, spectral power showed a low predictive capability in the prognostic model (Figure 5B). This may be due to the fact that we differentiated prognosis based on whether patients were EMCS or not, resulting in a relatively limited number of EMCS patients due to the high level of consciousness required.

Furthermore, this study has several limitations. Firstly, we did not consider the effect of individualized rehabilitation and medication in our analysis. Given the limited sample size, these variables could impact the accuracy of the results. Secondly, as previous research has shown, the complexity and methodological diversity of pDoC may lead to high heterogeneity in results (Ballanti et al., 2022). Larger sample size, multi-center studies, and more fine-grained classification schemes will contribute to explore more complex patterns of rehabilitation trajectories and improve the universality of the results. Specifically, integrating advanced analytical techniques from functional imaging into EEG data processing might facilitate a better understanding of how the brain’s dynamic functional connectivity evolves in pDoC. This could potentially lead to new insights into the neural mechanisms underlying pDoC and contribute to the development of more effective therapeutic strategies (Panda et al., 2023; Perl et al., 2023; Escrichs et al., 2022). Furthermore, a recent study has emphasized that deriving independent microstate atlases for subgroups can significantly elevate the Type I error rate, whereas applying a unified set of maps across the entire dataset can reduce such errors and enhance the reliability of the results (Murphy et al., 2024). In this study, given the particularity and complexity of pDoC, the use of a unified atlas for analysis could introduce additional errors, increasing the complexity of the analysis and the likelihood of erroneous conclusions. Therefore, we have followed the traditional analysis method (Baradits et al., 2020; Li et al., 2024; Sun et al., 2021). There is a need in the future to explore how to achieve a more scientific balance between the application of unified atlases and the differences among populations.

## 5 Conclusion

This study used multiple EEG patterns to represent the abnormal features of pDoC patients. These EEG features have demonstrated a certain degree of accuracy in predicting the 6-month prognosis of pDoC, with microstates making a significant contribution to the prognosis model. This innovative finding helps to understand the neurophysiological mechanisms behind consciousness recovery and provide additional insights to reveal the characteristics of brain signals in pDoC.

## Data Availability

The raw data supporting the conclusions of this article will be made available by the authors, without undue reservation.
